# Recent advances in bacteria‐based platforms for inflammatory bowel diseases treatment

**DOI:** 10.1002/EXP.20230142

**Published:** 2024-03-05

**Authors:** Jiaoying Lu, Xinyuan Shen, Hongjun Li, Juan Du

**Affiliations:** ^1^ Department of Gastroenterology The First Affiliated Hospital School of Medicine Zhejiang University Hangzhou China; ^2^ National Key Laboratory of Advanced Drug Delivery and Release Systems College of Pharmaceutical Sciences Zhejiang University Hangzhou China; ^3^ Department of Bioengineering University of California Los Angeles California USA; ^4^ Department of Hepatobiliary and Pancreatic Surgery, The Second Affiliated Hospital School of Medicine Zhejiang University Hangzhou Zhejiang China; ^5^ Liangzhu Laboratory Zhejiang University Hangzhou China

**Keywords:** diagnosis, drug delivery, engineered bacteria, inflammatory bowel diseases

## Abstract

Inflammatory bowel disease (IBD) is a recurring chronic inflammatory disease. Current treatment strategies are aimed at alleviating clinical symptoms and are associated with gastrointestinal or systemic adverse effects. New delivery strategies are needed for the treatment of IBD. Bacteria are promising biocarriers, which can produce drugs in situ and sense the gut in real time. Herein, we focus on recent studies of engineered bacteria used for IBD treatment and introduce the application of engineered bacteria in the diagnosis. On this basis, the current dilemmas and future developments of bacterial delivery systems are discussed.

## Graphical abstract

Recent studies on bacteria‐based platforms for the treatment of inflammatory bowel disease (IBD) through immunomodulation, antioxidative stress, barrier restoration, and microbial regulation are comprehensively reviewed. Current dilemmas and future development prospects of integrated IBD‐targeting bacterial platforms were also discussed.

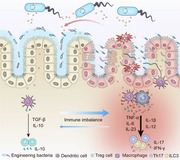

## 1 INTRODUCTION

With societal developments and lifestyle changes, inflammatory bowel diseases (IBDs) have become more common.^[^ ^1^ ^]^ IBDs are a series of recurrent chronic inflammatory diseases within the bowel, mainly including Crohn's disease (CD) and ulcerative colitis (UC). The specific etiology of IBD is still under investigation, but it is believed to be the combined result of genetic factors, alterations in the gut microbiota, abnormal immune responses, and environmental influences.^[^ ^2^ ^]^

Although there are rare cases with monogenetic defects and early onset, in which gene factors play a dominant role, most IBD patients are affected by multiple genes. Scientists have found over 200 risk alleles associated with IBD which are broadly related to immune response, autoimmunity, and autophagy.^[^ ^3^ ^]^ Western industrialized countries display the highest incidence rates of IBD worldwide while newly industrialized countries in Asia are experiencing a rising prevalence. This suggests that environmental factors, for example, diet and stress, potentially contribute to the etiology and progression of IBD. Specifically, dietary fiber intake may be linked to a lower risk of CD and long‐term consumption of trans‐unsaturated fatty acids may increase the incidence of UC, according to several large prospective cohort studies.^[^ ^4^ ^]^ Researchers found that environmental factors could disrupt the composition of the gastrointestinal microorganism to eventually promote the pathogenesis of IBD. Gastrointestinal bacteria have a tight and complex relation with the gut immune system. They promote the maturation of the gastrointestinal immune system and maintain the integrity of the intestinal barrier.^[^ ^5^ ^]^ In IBD patients, a reduced diversity of microorganisms and metabolites was observed.^[^ ^6^ ^]^ *Blautia*, *Faecalibacterium*, and *Ruminococcus* were discovered to be keystone taxa in IBD, but with more association in CD, by defining distinct networks of taxa associations within intestinal biopsies.^[^ ^7^ ^]^ In addition to defective biological barriers, chemical, and mechanical barriers are also impaired in IBD. A protective chemical barrier is formed by the mucus secreted by goblet cells and antimicrobial peptides (AMPs) generated mainly by Paneth cells.^[^ ^8^ ^]^ Bacteria cannot penetrate the dense and thick inner colon mucus in normal humans, but can pass the thinner colon mucus and reach the epithelium in active UC.^[^ ^9^ ^]^ The integrity of the physical barrier is attributed to the intestinal epithelial cells and the apical tight junctions between adjacent epithelial cells. However the tight junction is reduced and the expression of the transmembrane proteins is abnormal in IBD.^[^ ^10^ ^]^

The gastrointestinal tract is the biggest immune organ with the innate, adaptive, and mucosal immune system. Innate immune cells (ICCs), including macrophages, dendritic cells, and innate lymphoid cells mediate the host immune response. They express pattern recognition receptors to recognize pathogens and trigger immune responses. ICCs orchestrate immune tolerance with gut commensal microbiota and provide a defense against exogenous pathogens. Through secretion of cytokines and antigen presentation, ICCs can trigger the adaptive immune system to generate specific and robust immune responses.^[^ ^11^ ^]^ It is now believed that innate and adaptive immunity are equally important in IBD. Adaptive immune cells, especially T cells, migrate to the gut to initiate and maintain the immune response. T cells are broadly classified into two types: proinflammatory T cells and anti‐inflammatory T cells. Inflammatory T cells include CD8+ cytotoxic T cells and CD4+ T helper (Th) cells, while regulatory T cells (Tregs) are anti‐inflammatory T cells.^[^ ^12^ ^]^ Mounting evidence indicates that immunological dysregulation leading to inflammation is a vital contributor to IBD.^[^ ^13^ ^]^ Infiltration of inflammatory T cells and excessive inflammatory cytokines are the common denominator of IBD. The mucosa in IBD patients has an unbalanced ratio of proinflammatory and anti‐inflammatory T cells. Th1, Th2, and Th17 inflammatory pathways are activated, and inflammatory cytokines increased, including interleukin‐1 (IL‐1), interleukin‐6 (IL‐6), interleukin‐13 (IL‐13), interleukin‐17 (IL‐17), interleukin‐22 (IL‐22), tumor necrosis factor‐α (TNF‐α), interferon‐γ (IFN‐γ).^[^ ^2^ ^]^ However, there are some differences in the specific T cells and cytokines involved between CD and UC. Th1 cells secret TNF‐α and IFN‐γ that promote the pathophysiology of CD, while UC is associated with atypical Th2 immune response, characterized by IL‐13 releasing.^[^ ^14^ ^]^ However, the efficacy of IFN‐γ or IL‐13 specific antibodies was not established in IBD patients clinically.^[^ ^15^ ^]^ The manifestations of UC are repeated episodes of bloody diarrhea and abdominal pain, usually accompanied by fever in the active period. Long‐standing UC patients have an increased risk of colon cancer.^[^ ^16^ ^]^ CD is a chronic inflammatory granulomatous disease characterized by recurrent abdominal pain, diarrhea, and weight loss, often complicated by strictures, fistulae, and abscesses. The traditional treatment for IBD is mesalazine/5‐aminosalicylic acid (5‐ASA) and glucocorticoids. Advanced therapeutic interventions include mercaptopurine analogs and immunosuppressive biological agents. These drugs are effective in alleviating inflammation and greatly reduce the rate of disability and surgery for IBD. 5‐ASA is the primary therapeutic option for mild‐to‐moderate IBD, serving as an effective approach for both induction and maintenance of remission, with minimal occurrence of treatment‐associated adverse incidents.^[^ ^17^ ^]^ Nevertheless, the efficacy of 5‐ASA is limited when dealing with patients experiencing moderate‐to‐severe IBD, and its effectiveness is inconsistent in certain individuals presenting with mild‐to‐moderate IBD. For other agents, the side effects of their long‐term use cannot be ignored. Gastrointestinal adverse effects such as nausea, vomiting, and loss of appetite are common with immunosuppressive agents, and the use of immunosuppressive and biological agents increases the incidence of opportunistic infections. Patients on thiopurine treatment have increased risks of lymphoproliferative disorders, myeloproliferative disorders, non‐melanoma skin cancer, and cervical high‐grade dysplasia and cancer.^[^ ^18^ ^]^ Besides, glucocorticoids are less recommended for long‐term use due to the disruption to the body's metabolism, including sugars, proteins, fats, electrolytes, osteoporosis and phosphorus, peptic ulcers, and lower immunity.^[^ ^19^ ^]^

It is evident that IBDs necessitate the exploration of novel therapeutic interventions that mitigate systemic adverse effects. In recent years, significant advancements in therapy approaches have emerged, encompassing novel pharmacological agents and innovative drug‐delivery mechanisms, which aim to tackle the challenges associated with IBDs.^[^ ^20^ ^]^ Bacteria exhibit considerable potential as carriers for targeted drug delivery to inflamed lesions, leading to increased localized drug concentrations, improved therapeutic outcomes, and reduced systemic side effects. Bacteria have been utilized as therapeutic agents for centuries owing to their close relation with the human internal environment.^[^ ^21^ ^]^ Probiotics have exhibited therapeutic potential in the management of specific medical conditions, particularly for intestinal disorders such as irritable bowel syndrome,^[^ ^22^ ^]^ diarrhea,^[^ ^23^ ^]^ constipation,^[^ ^24^ ^]^ and UC.^[^ ^25^ ^]^ With a better understanding of bacteria biology and the maturing technology of bacterial gene editing, researchers have been able to transform probiotics into bio‐factories to produce various bioactive. For instance, Steidler et al. used *Lactococcus lactis* to secrete IL‐10 for colitis treatment in mice and achieved remarkable results.^[^ ^26^ ^]^ Although no significant difference was seen in later clinical trials compared to a placebo, it provided the idea of using probiotics to deliver drugs to the gut and gave rise to subsequent studies.

**FIGURE 1:**
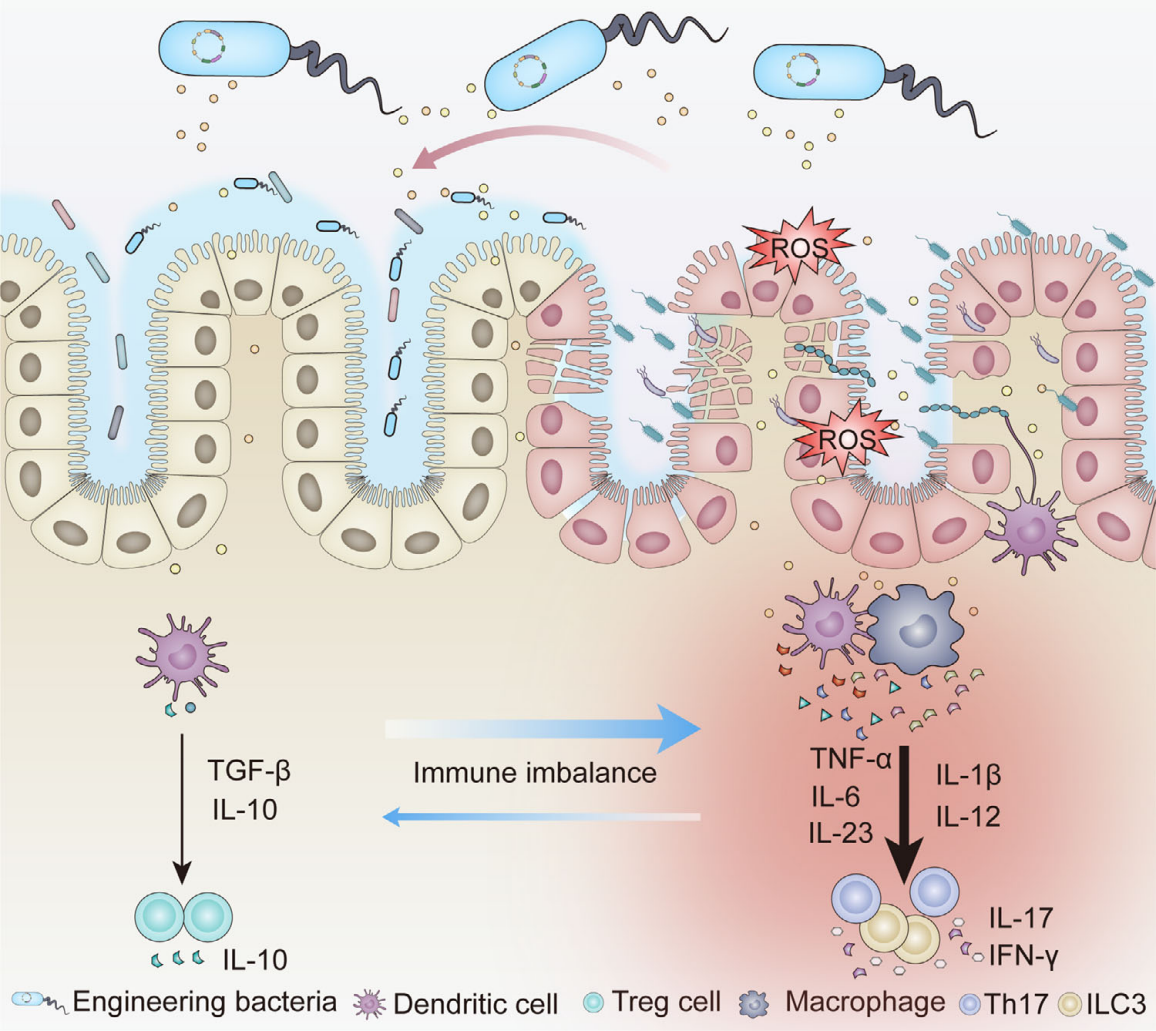
Schematic illustration of engineered bacteria that generate therapeutic agents for inflammatory bowel disease. Engineered bacteria generate therapeutic agents to treat IBD through immunomodulation, antioxidative stress, barrier restoration, and microbial regulation in the intestine.

In this review, we first present recent studies on bacteria‐based platforms for the treatment of IBD through four mechanisms: immunomodulation, antioxidative stress, barrier restoration, and microbial regulation (Figure 1). Then we introduce bacterial detection of intestinal disorders and discuss current dilemmas and future development prospects at the end of the review.

## 2 BACTERIA PLATFORMS FOR IBD DRUG DELIVERY

The gastrointestinal tract, serving as a pivotal organ for digestion and nutrient absorption, possesses formidable digestive and catabolic functions, posing a challenge to the effective oral administration of biologic drugs. Notably, the presence of gastric juices, characterized by potent acidic and enzymatic properties, presents a big hurdle, leading to the inactivation of a majority of biological drugs.^[^ ^27^ ^]^ The stomach is considered a natural antimicrobial barrier due to its harsh luminal environment. However, certain enteropathogenic bacteria can survive the strongly acidic gastric juices, potentially triggering intestinal infections. A clinical trial in 2006 demonstrated that the yogurt bacteria could be detected in the feces of healthy individuals who consumed yogurt, indicating the resilience of probiotics within the gastrointestinal tract.^[^ ^28^ ^]^ Early studies have demonstrated that the lactic acid bacteria can endure gastric juices and adhere to intestinal cells.^[^ ^29^ ^]^ Bacteria have evolved diverse acid‐resistant systems to enhance their survival in acidic environments.^[^ ^30^ ^]^ There are significant variations in the survival capabilities of different bacteria in acidic conditions. In addition to screening bacteria with high survival rates in the gastrointestinal tract as vectors, bacteria can also be encapsulated to further enhance their viability.^[^ ^31^ ^]^ Traditional drug‐delivery materials such as sodium alginate, chitosan, hydrogels, and emerging nano‐delivery systems are utilized for the encapsulation of probiotics.^[^ ^32^ ^]^ Additionally, the survival rate of bacteria is influenced by the dosage form and formulation. For example, drying techniques and tablets can improve the acid tolerability of probiotic bacteria.^[^ ^33^ ^]^ It is important to note that intraperitoneal and intravenous injections are also viable methods for administering engineered bacteria‐based delivery systems, particularly in treating tumors and non‐gastrointestinal diseases.^[^ ^34^ ^]^ However, due to the relative sterility of the abdominal cavity and bloodstream, these administration routes necessitate heightened caution. This careful approach is essential to balance the desired therapeutic effects with the potential risks of bacterial infection.

The intestine, as an organ inherently colonized by bacteria, bestows upon bacteria several specific advantages when compared to other delivery systems. Beyond the customary benefits associated with drug delivery, such as oral administration, minimized systemic side effects, and the transport of macromolecules, bacteria offer unique features, including intestinal targeting, in situ biologics production, intestinal colonization, and modulation of intestinal flora. Intestinal mucus is one more factor that influences the effect of drug absorption.^[^ ^27^ ^]^ However, in the case of IBD, the impairment of the epithelial barrier, including the mucus layer, at the lesion site renders both drugs and bacteria more accessible to exert their effects. Furthermore, the heightened presence of vessels and chemoattractants secreted by necrotic cells in the inflamed area increases the likelihood of bacteria targeting the lesion due to their chemotactic properties.^[^ ^35^ ^]^ Moreover, intestinal colonization not only facilitates the regulation of intestinal flora, and competitive marginalization of pathogens but also supports long‐term in situ drug delivery and relatively stable drug release. This characteristic reduces the frequency of drug administration, thereby enhancing patient compliance.

Decades of development in bacteria‐based therapeutics proved that the risk of administering bacteria within the human body could be circumvented with probiotic bacteria. For instance, lactic acid bacteria and *Escherichia coli* Nissle 1917 (EcN) are the two most commonly used probiotics. Many lactic acid bacteria, including *Lactococcus*, *Lactobacillus*, *Bifidobacterium*, and *Streptococcus*, are recognized as safe by the US Food and Drug Administration.^[^ ^36^ ^]^ Among them, *L. lactis* is the most reliable strain for genetic modification. *L. lactis* has potential anti‐colitis properties^[^ ^37^ ^]^ and can survive throughout the digestive tract,^[^ ^38^ ^]^ which are some of the reasons for its use in IBD drug delivery. *E. coli* Nissle 1917 (EcN) is a non‐pathogenic *E. coli* with serotype O6:K5:H1, which was isolated by researcher Alfred Nissle in 1917.^[^ ^39^ ^]^ Owing to its genetic stability and absence of specific virulence factors, EcN is widely regarded as a safe probiotic strain and has a long‐standing track record of over a century in human health applications. EcN's flagella are not only key adhesion factors but also are considered to be related to hardening the epithelial barrier to prevent the adherence and invasion of other microbes.^[^ ^40^ ^]^ In several clinical trials, EcN has been proven to be as effective as mesalazine in UC treatment.^[^ ^41^ ^]^ Therefore, using these probiotics as a drug‐delivery vehicle can get a dual therapeutic effect. Adding to the ease of genome editing and the availability of a common molecular biology toolbox, *L. lactis* and EcN are matured platforms for drug delivery to the colon. Some other bacteria, like *Bifidobacterium longum*,^[^ ^42^ ^]^ *Salmonella typhimurium*,^[^ ^43^ ^]^ and *Clostridium butyricum*,^[^ ^44^ ^]^ are also used for drug delivery in IBD.

## 3 IN SITU GENERATION OF THERAPEUTIC AGENTS BY BACTERIA‐BASED PLATFORMS

The stomach and small intestine can digest a wide range of substances, so it is difficult for drugs such as proteins and peptides to reach the lower gastrointestinal tract to act directly and locally. Therefore, most bioactive, like proteins and peptides can only be administered intravenously. Engineered bacteria can pass through the gastrointestinal tract, and then produce bioactive in situ, while colonizing the gut and then exerting long‐term effects. This exemption from digestive degradation frees up the types of biological agents, giving IBD treatment a better chance of being closer to etiological treatment. A summary of bacteria‐based platforms for IBD is summarized in Table 1.

**TABLE 1:** Summary of bacterial delivery for drug in situ generation for inflammatory bowel disease.

Mechanism of function	Bacterial vectors	Therapeutic agents	IBD Models	Ref.
Immunomodulatory bioactive	EcN, *Lactococcus lactis*	IL‐10	DSS‐colitis mice, IL‐10(‐/‐) mice, TNBS‐induced colitis mice	[26, 45]
	*L*. *lactis*	IL‐27	CD4+ CD45RBhi T cell transferred Rag‐/‐ mice enterocolitis model, DSS‐colitis mice	[46]
	*L*. *lactis*, *Escherichia coli*	IL‐35	DSS‐colitis mice	[47]
	EcN	IFNL1	Caco‐2/Jurkat T cell co‐culture model, scaffold‐based 3D co‐culture model	[48]
	*L*. *lactis*	SlpA	CD4+ CD45RBhi T cell transferred Rag‐/‐ mice enterocolitis model	[49]
	*L*. *lactis*	Bovine lactoferricin‐lactoferrampin	DSS‐colitis mice	[50]
	EcN	Sj16	DSS‐colitis mice	[51]
	EcN	3HB	DSS‐colitis mice	[52]
Proinflammatory cytokine antibodies/antagonists	*L*. *lactis*	Monovalent and bivalent murine TNF‐neutralizing nanobodies	DSS‐colitis mice	[53]
	*Bifidobacterium longum, L*. *lactis, Lactobacillus salivarius*	TNF‐α antibodies/antagonists	(C57/BL6 × DBA/2) F1 hybrid mice, /	[54]
	*L. salivarius*	Anti‐IL‐17A fynomer	/	[54c]
	*L. Salivarius, L*. *lactis*	IL‐23 antibodies/antagonists	/	[54, 55]
	*L*. *lactis*	Anti‐IL‐6 affibody ZIL	/	[56]
	*L*. *lactis*	IL‐1Ra	DSS‐colitis mice	[57]
Enzyme of anti‐inflammatory agents	*Lactobacillus paracasei*	NAPE‐PLD	DSS‐colitis mice	[58]
	EcN	Butyryl‐CoA dehydrogenase	DSS‐colitis mice	[59]
Antioxidant agents/enzymes	*L*. *lactis*	BPC‐157	149BR fibroblast cell	[60]
	*B*. *longum*	MnSOD	LPS‐induced inflammatory cell, DSS‐colitis mice	[42]
	EcN	CAT and SOD	DSS‐colitis mice, TNBS‐induced colitis mice, oxazolone‐induced IBD mice	[61]
Barrier restorative function	*Lactobacillus reuteri*	hIL‐22	Human intestinal enteroid	[62]
	EcN	Trefoil factor	DSS‐induced colitis	[63]
	*Clostridium butyricum*	pEGF	IPEC‐J2	[44]
	EcN	Human EGF	DSS‐colitis mice	[64]
Microbial regulation	*L*. *lactis*	Cathelicidin	DSS‐colitis mice	[65]
	*L*. *lactis*	Lactoferricin‐lactoferrampin	DSS‐colitis mice	[50]
	*L*. *lactis*	PAP	5‐FU‐treated mucositis mice	[66]
	*L*. *lactis*	mBD14	DSS‐colitis mice	[67]

Abbreviations: 3HB, (R)−3‐hydroxybutyrate; CAT, catalase; DSS, dextran sodium sulfate; IFNL1, interferon lambda 1; IPEC‐J2, porcine intestinal epithelial cell line; LPS, lipopolysaccharide; mBD14, mouse β‐defensin 14; MnSOD, manganese superoxide dismutase; NAPE‐PLD, *N*‐acylphosphatidylethanolamine‐preferring phospholipase D; PAP, pancreatitis‐associated protein; pEGF, porcine epidermal growth factor; Sj16, schistosome immunoregulatory protein; SlpA, surface layer protein A; SOD, superoxide dismutase; TNBS, 2,4,6‐trinitrobenzene sulfonic acid.

### 3.1 Immunomodulation

Although the exact etiology of IBD is still not clear, immune imbalance is the key part of IBD. Therefore, delivery agents to modify gut immunity are a major idea in the studies of engineered bacteria for IBD.

#### 3.1.1 Immunomodulatory bioactives

**FIGURE 2:**
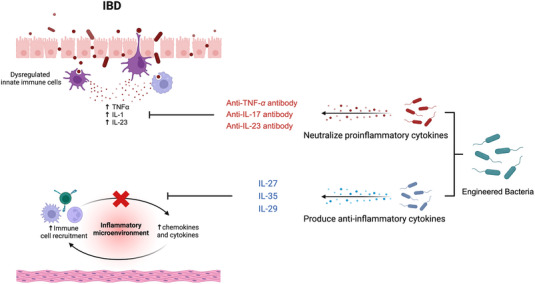
Schematic illustration of engineered bacteria that achieved immunomodulation in inflammatory bowel disease.

The early representative study treated colitis mice with engineered *L. lactis‐*secreting IL‐10, finding that in situ generation of engineered bacteria can lower the therapeutic dose of IL‐10.^[^ ^26^ ^]^ In recent years, researchers have attempted to produce other anti‐inflammatory cytokines such as IL‐27,^[^ ^46^ ^]^ IL‐35,^[^ ^47^ ^]^ and interferon lambda 1 (IFNL1 or IL‐29)^[^ ^48^ ^]^ by modified bacteria (Figure 2). Whether administered post‐model^[^ ^47b^ ^]^ or preventatively,^[^ ^47a^ ^]^ a reduction in Th17 cells and an increase in Treg cells were observed in mice treated with IL‐35‐expressing bacteria. In murine enterocolitis,^[^ ^46^ ^]^ oral delivery of IL‐27‐secreting *L*. *lactis* (LL‐IL‐27) improved survival significantly. A disease activity index (DAI) is a commonly used metric to assess disease activity in IBD mouse models, consisting of three parts: blood in feces, stool consistency, and weight loss. LL‐IL‐27–treated mice show a decreased DAI and histological damage compared with untreated and *L*. *lactis* control–treated mice. Researchers further compared the treatment of LL‐IL‐27 with systemic treatment with recombinant mouse IL‐27 (rm IL‐27). LL‐IL‐27 reduced the DAI by about 50% and eliminated microscopic lesions, while systemic IL‐27 had little therapeutic effect. Further research addressed the anti‐inflammatory mechanism of LL‐IL‐27, including reducing inflammatory cytokines and increased IL‐10, deceased CD4+ T cells, and lower CD4/CD8 ratio. Additionally, researchers found that systemic administration of IL‐27 greatly increased the level of plasma IL‐10 but had a lower level of IL‐10 in the distal colon compared with mice receiving LL‐IL‐27, demonstrating the advantage of topical delivery of bacteria.

In addition to cytokines, there are other bioactive inhibiting inflammation, such as bovine lactoferricin‐lactoferrampin,^[^ ^50^ ^]^ surface layer protein A,^[^ ^49^ ^]^ (R)−3‐hydroxybutyrate,^[^ ^52^ ^]^ and schistosome immunoregulatory protein (Sj16),^[^ ^51^ ^]^ which could be generated by bacteria in the gut and then protect mice from colitis.

#### 3.1.2 Proinflammatory cytokine inhibitors/antagonists

Neutralization of proinflammatory cytokines is a common strategy for IBD. TNF‐α is a key proinflammatory cytokine in the pathogenesis of IBD, several anti‐TNF‐α agents have been approved with remarkable success.^[^ ^68^ ^]^ As all anti‐TNF‐α agents currently in clinical use are administered intravenously or subcutaneously, the development of new oral anti‐TNF‐α agents is a popular direction. Researchers have developed various methods to deliver anti‐TNF‐α with bacteria, such as bacteria autonomously secreting anti‐TNF nanobody^[^ ^53^ ^]^ or scFv,^[^ ^54a^ ^]^ or displaying TNF‐α‐binding affibody on the surface.^[^ ^54^, ^69^ ^]^ These methods reduced inflammation in colitis mice, but the therapeutic effect of TNF‐α‐binding *L. lactis* did not differ significantly from control *L. lactis*.^[^ ^69^ ^]^ It is also worth mentioning that there is a genetic intervention targeting TNF‐α. With the help of invasive proteins^[^ ^70^ ^]^ or bacterial internalization by eukaryotic cells, gene fragments targeting TNF can be transferred into the colonic epithelium. Additionally, researchers have designed bacteria that express proteins to block IL‐17 and IL‐23.^[^ ^54^, ^55^ ^]^ Using LysM repeats as a cell wall anchor, cytokine‐binding non‐Ig scaffolds (anti‐IL‐17A fynomer, anti‐IL‐23‐binding adnectin and anti‐TNF‐α‐binding affibody) can be anchored on the surface of bacteria. These proteins were expressed in fusion with the secretion signal (Usp45) and LysM‐containing cell wall anchor in *L. lactis*.^[^ ^54c^ ^]^ Similarly, anti‐IL‐6 affibody anchored on the surface of *L. lactis* by fusing with Usp45 and anchor protein AcmA, could capture IL‐6 specifically from cell culture supernatant.^[^ ^56^ ^]^ Another way to prevent inflammatory pathways is to antagonize proinflammatory cytokine receptors. Plavec et al. transformed *L. lactis* with plasmids containing the cDNA sequences coding for REX protein, which has been reported as an IL‐23 receptor (IL‐23R) antagonist.^[^ ^55b^ ^]^ They confirmed that the IL‐23R antagonists produced by *L. lactis* could efficiently bind with the IL‐23R‐IgG chimera. Later, Namai et al. designed a modified *L. lactis* hypersecreting IL‐1Ra, which can decrease the colitis severity in mice after oral administration.^[^ ^57^ ^]^

#### 3.1.3 Anti‐inflammatory enzymes

Another strategy is to use engineered bacteria to generate drug synthases, which can also achieve in situ drug manufacturing in the colon. Despite palmitoylethanolamide (PEA) being a safe naturally anti‐inflammatory agent,^[^ ^71^ ^]^ therapeutic colonic concentrations following oral treatment are challenging to reach. PEA is derived from the hydrolysis of its phospholipid precursor, *N*‐acylphosphatidylethanolamine‐specific phospholipase D (NAPE‐PLD). Engineered NAPE‐PLD‐expressing *Lactobacillus paracasei* can colonize in the colon and manufacture PEA under ultra‐low palmitate supply and minimize the extent of disease in dextran sodium sulfate (DSS)‐induced colitis mice.^[^ ^58^ ^]^

Butyric acid is a short‐chain fatty acid that serves as an energy source for the colonic epithelium as well as having anti‐inflammatory, epithelial barrier maintenance, and immunomodulatory properties.^[^ ^72^ ^]^ As butyric acid cannot reach the colon by oral administration, Park and his colleagues cloned and expressed the butyric acid‐producing gene butyryl‐CoA dehydrogenase in EcN, which was able to manufacture butyric acid in the colon and prevent tissue damage in mice with DSS‐induced colitis.^[^ ^59^ ^]^

### 3.2 Anti‐oxidation

**FIGURE 3:**
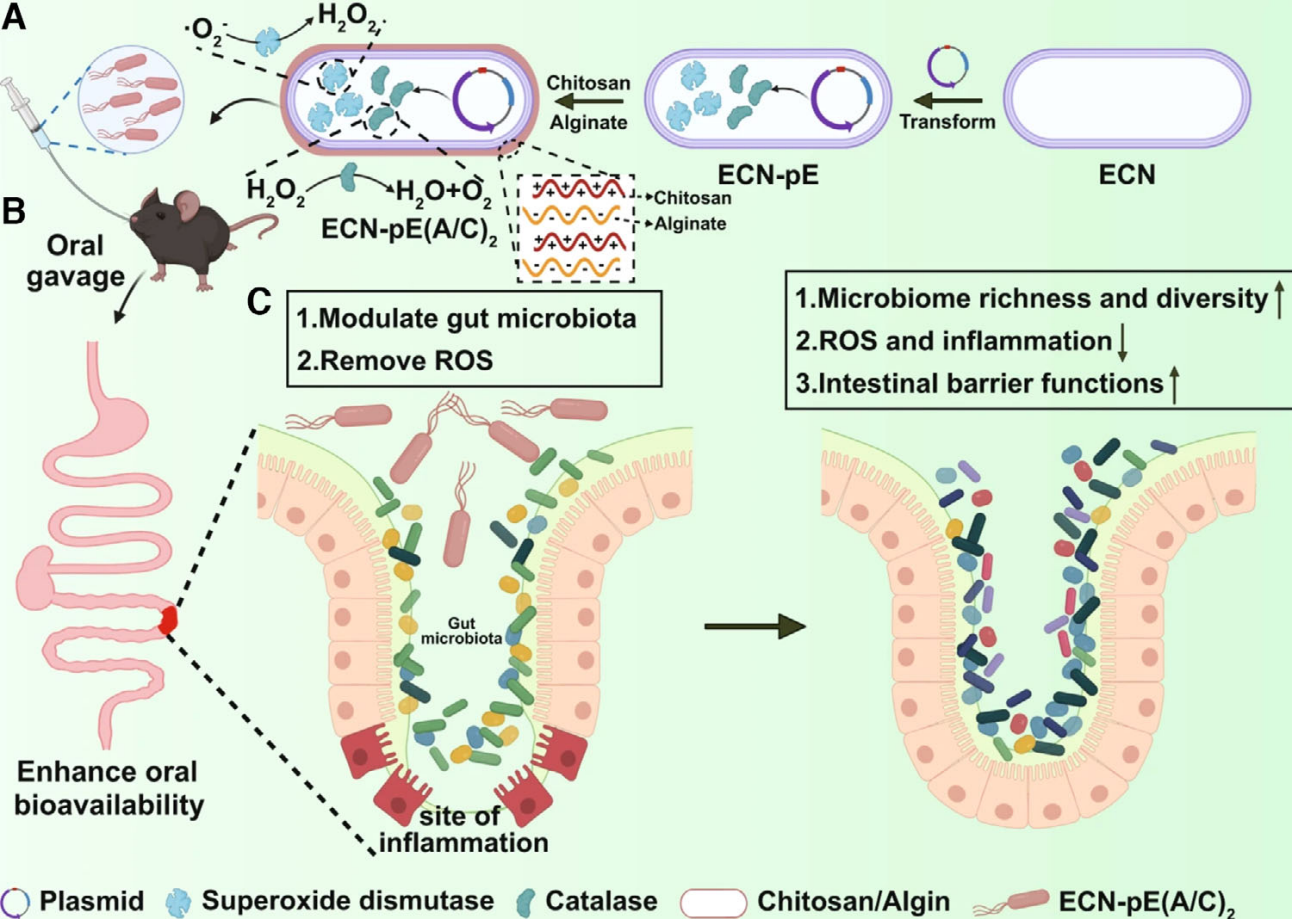
Engineer probiotic with antioxidant enzymes for inflammatory bowel disease treatment. (A) *Escherichia coli* Nissle 1917 (ECN) was engineered to produce catalase (CAT) and dismutase (SOD). Then, chitosan and sodium alginate were used to encapsulate the ECN via layer‐by‐layer (ECN‐pE(C/A)2), (B) which significantly elevated its oral bioavailability. ECN‐pE, ECN (pET28a‐T5‐CAT‐SOD). (C) ECN‐pE(C/A)2 relieves inflammatory bowel disease by removing reactive oxygen species and modulating the intestinal flora. Reproduced under the terms of the Creative Commons CC BY license.^[^ ^61^ ^]^ Copyright 2022, Zhou et al.

Since increased reactive oxygen species (ROS) are frequently observed in many IBD patients and colitis mice, there is substantial evidence showing that oxidative stress plays a key role in the IBD.^[^ ^73^ ^]^ Several studies have employed nanoparticles as carriers for delivering antioxidant drugs, yielding promising outcomes in the treatment of mice with colitis.^[^ ^74^ ^]^ Hence, one strategy employing bacteria involves the localized secretion of antioxidants, such as gastric pentadecapeptide BPC‐157, achieved through genetically engineered probiotics within the colon.^[^ ^60^ ^]^ An alternative approach is the secretion of enzymes aimed at clearing ROS.^[^ ^75^ ^]^ Manganese superoxide dismutase (MnSOD), a pivotal antioxidant enzyme capable of neutralizing various ROS,^[^ ^76^ ^]^ poses a challenge due to its substantial molecular size. To overcome this hurdle, Liu et al. ingeniously fused the MnSOD gene with PEP‐1, a penetratin facilitating the cellular transfer of SOD. The resulting *Bifidobacterium* carrying the PEP‐1‐hMnSOD fusion gene demonstrated successful MnSOD expression in both an LPS‐induced inflammatory cell model and DSS‐induced colitis mice.^[^ ^42^ ^]^ In a related initiative, Zhou et al. modified EcN to produce catalase (CAT) and superoxide dismutase (SOD) (Figure 3).^[^ ^61^ ^]^ By coating the bacteria with two layers of chitosan/sodium alginate through a layer‐by‐layer process (ECN‐pE(C/A)2), the oral utilization of the bacteria significantly increased. These modified bacteria exhibited therapeutic efficacy in various mice models of colitis, manifesting in alleviated IBD symptoms, reduced inflammation levels, colonic epithelial repair, and improved intestinal flora.

### 3.3 Barrier restoration

**FIGURE 4:**
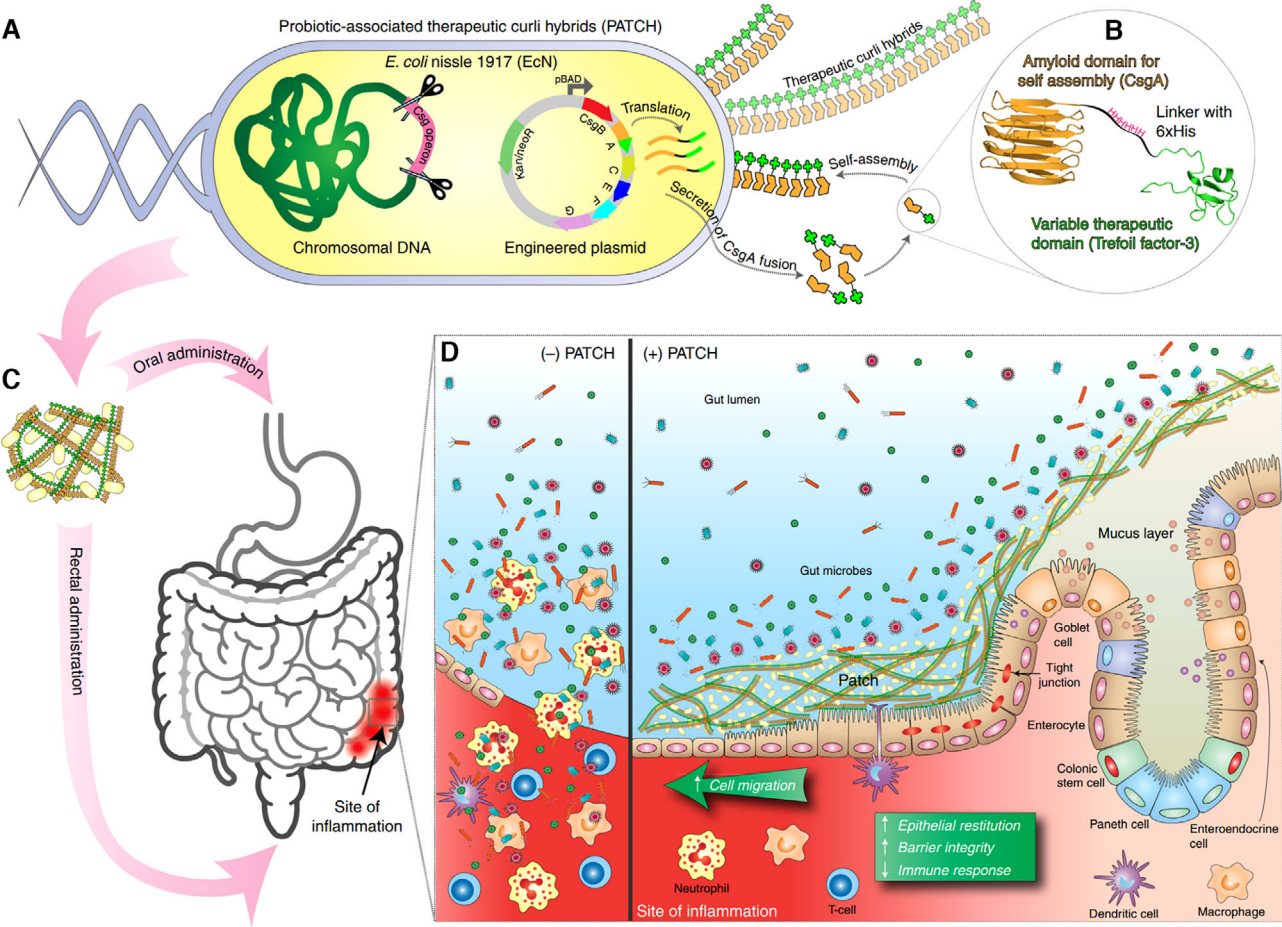
Production and application of probiotic‐associated therapeutic curli hybrids (PATCH). (A) *Escherichia coli* Nissle 1917 (EcN) contains plasmids encoding a synthetic curli operon while simultaneously eliminating the csg operon from its chromosomal DNA. The genetic modification facilitated the synthesis of chimeric CsgA proteins, which can self‐assemble extracellularly to form therapeutic curli hybrid fibers after secretion. (B) CsgA (yellow) was genetically linked to trefoil factor‐3 (bright green) via the flexible linker (black) including a 6xHis tag. (C) Engineered bacteria are mass produced before being given to the subject orally or rectally. (D) Inflammatory lesions in inflammatory bowel disease cause colonic crypt structure loss, epithelial tissue destruction, and impaired barrier integrity, which result in infiltration of luminal contents and immune cells (left panel, (‐) PATCH). The utilization of PATCH improves inflammatory bowel disease activity (right panel, (+) PATCH). Reproduced under the terms of the Creative Commons CC BY license.^[^ ^79^ ^]^ Copyright 2019, Praveschotinunt et al.

In IBD, impaired intestinal barrier function increases mucosal permeability, allowing bacteria and antigens to penetrate the epithelium and exacerbate inflammation. IL‐22 is thought to be a major regulator of the intestinal mucosal barrier and wound healing. Ortiz‐Velez found that the production of human IL‐22 in *Lactobacillus reuteri* can be maximized through optimization of the secretory signal sequence and triggering the production of AMPs in human enteroids.^[^ ^62^ ^]^ Trefoil factor (TFF) and epidermal growth factor (EGF) are peptides that regulate intestinal epithelium healing in the body.^[^ ^77^ ^]^ Curli nanofibers engineered from *E. coli* can construct a self‐renewing hydrogel that works as a mucosal adhesive to extend gut residency time.^[^ ^78^ ^]^ By using the curli secretion systems, researchers designed EcN strains capable of secreting the monomer unit of curli fibers (CsgA). These CsgA monomer units were fused with specific TFFs, resulting in the formation of a curli fiber matrix combined with TFF molecules (Figure 4).^[^ ^79^ ^]^ The engineered EcN enemas enhanced protection against DSS‐induced colitis in mice and are associated with mucosal restoration and immune regulation. Despite there being no difference in therapy between tethered and untethered TFF3, it is still worthwhile to investigate the role of tethered therapies for their local retention effect.

Analysis of the IBD patient data sets revealed that the expression of the EGF receptor (EGFR) was reduced in IBD patients compared to the healthy population.^[^ ^64^ ^]^ EGF enemas have been proven effective in UC in a clinical trial.^[^ ^80^ ^]^ Following the application of EGF‐secreting recombinant bacteria, there is an upregulation in levels^[^ ^44^ ^]^ or phosphorylation of EGFR.^[^ ^64^ ^]^ EGFR inhibitors attenuate the therapeutic effect of EGF‐EcN, demonstrating its effectiveness in reverse.^[^ ^64^ ^]^ Local EGF secretion in the gut minimizes the risk of extraintestinal tumor formation. Furthermore, repeated treatment of EGF‐EcN did not result in intestinal abnormal proliferation in the AOM/DSS–induced cancer model, which might be associated with reduced epithelial damage.^[^ ^64^ ^]^

Outer membrane vesicles (OMVs) are natural nanoparticles released by bacteria.^[^ ^81^ ^]^ OMVs secreted by the intestine colonizing bacteria can pass through the gut epithelial barrier and interact with immune cells in the lamina propria.^[^ ^82^ ^]^ By gene editing, OMVs could also express therapeutic proteins.^[^ ^83^ ^]^ Some studies have found the role of OMVs in the treatment of colon cancer, with medications supplied by OMVs being more aggregated in the colon and having a lower influence on other organs such as the liver and kidneys.^[^ ^84^ ^]^ Keratinocyte growth factor‐2 (KGF‐2) is a human therapeutic protein that promotes epithelial cell proliferation. After cloning the KGF‐2 gene downstream the major outer membrane protein OmpA, KGF‐2 is contained within the lumen of OMVs, which then can be taken orally and reduce the colon inflammation in DSS‐induced colitis mice.^[^ ^83^ ^]^

### 3.4 Microbial regulation

The distal ileum and colon contain a large number of microorganisms that interact with the host. IBD patients' intestinal flora is altered. In IBD patients, there is a notable rise in facultative anaerobes alongside a decline in obligate anaerobes.^[^ ^6^ ^]^ Moreover, there is an increase in inflammatory‐competent bacteria, while bacteria with anti‐inflammatory‐competent experience a reduction.^[^ ^2^ ^]^ There have been clinical trials to employ fecal microbiota transplant (FMT) to treat IBD,^[^ ^85^ ^]^ Fang et al. showed a pooled rate of clinical remission of 28.8% and clinical response of 53%.^[^ ^86^ ^]^ FMT is currently regarded to be a safe and effective therapy for recurrent *Clostridioides difficile* infection (CDI),^[^ ^87^ ^]^ which also showed promising results in IBD patients with CDI.^[^ ^88^ ^]^ Despite of the low occurrence of adverse events, there are still risks that cannot be overlooked. As complex mixtures of living organisms, the transplant may be mixed with pathogenic or antibiotic‐resistant microorganisms.^[^ ^89^ ^]^ Engineered bacteria provides a method to avoid the risks. The gut itself can regulate flora by secreting AMPs.^[^ ^8^ ^]^ Researchers have successfully regulated gut microbial through genetically modified bacteria to secrete AMPs, such as cathelicidin,^[^ ^65^, ^90^ ^]^ bovine lactoferricin‐lactoferrampin,^[^ ^50^ ^]^ and mouse β‐defensin 14 (mBD14).^[^ ^67^ ^]^ Cathelicidin delivered by modified *L. lactis* demonstrated better efficacy in clinical symptoms and histology injuries than normal *L. lactis*. As a reference drug, sulfasalazine only reduced clinical symptoms but not the colon mucosal damage. Regrettably, they did not detect the changes in flora in the mice to validate the flora regulation effect.^[^ ^65^, ^90^ ^]^ With *L. lactis* delivery of lactoferricin‐lactoferrampin, the gut dysbiosis induced by DSS was reversed.^[^ ^50^ ^]^ Similarly, oral delivery of mBD14‐producing *L*. *lactis* notably increased the abundance of *Faecalibacterium prausnitzii* and *Akkermansia muciniphila*, two probiotic bacteria previously found to be reduced in UC patients, and prevented aberrant enrichment of *E*. *coli* in colitis mice.^[^ ^67^ ^]^ Pancreatitis‐associated protein (PAP) is also an important type of AMP. After delivery by bacteria, 16S rDNA sequencing showed an increased butyric acid‐producing bacteria and reduced potential opportunistic bacteria in a mouse model of mucositis.^[^ ^66^, ^91^ ^]^

## 4 BACTERIA FOR THE DIAGNOSIS OF INTESTINAL DISEASES

Bacterial medical systems can be employed not only for therapy but also for diagnosis. In gastrointestinal imaging, a study internalized ultrasmall (1–2 nm) hafnia nanoparticles into probiotics to achieve gastrointestinal tract delivery, which resulted in larger contrast concentrations and better imaging.^[^ ^92^ ^]^ Invasive diagnostics are frequently used in the clinical diagnosis of IBD. Some scientists leverage engineered bacteria to detect inflammatory signals and convert them into easily discernible signs. When the smart bacteria are taken into the gut, we will be able to assess the state of intestinal inflammation when the bacteria are excreted with the feces.

**FIGURE 5:**
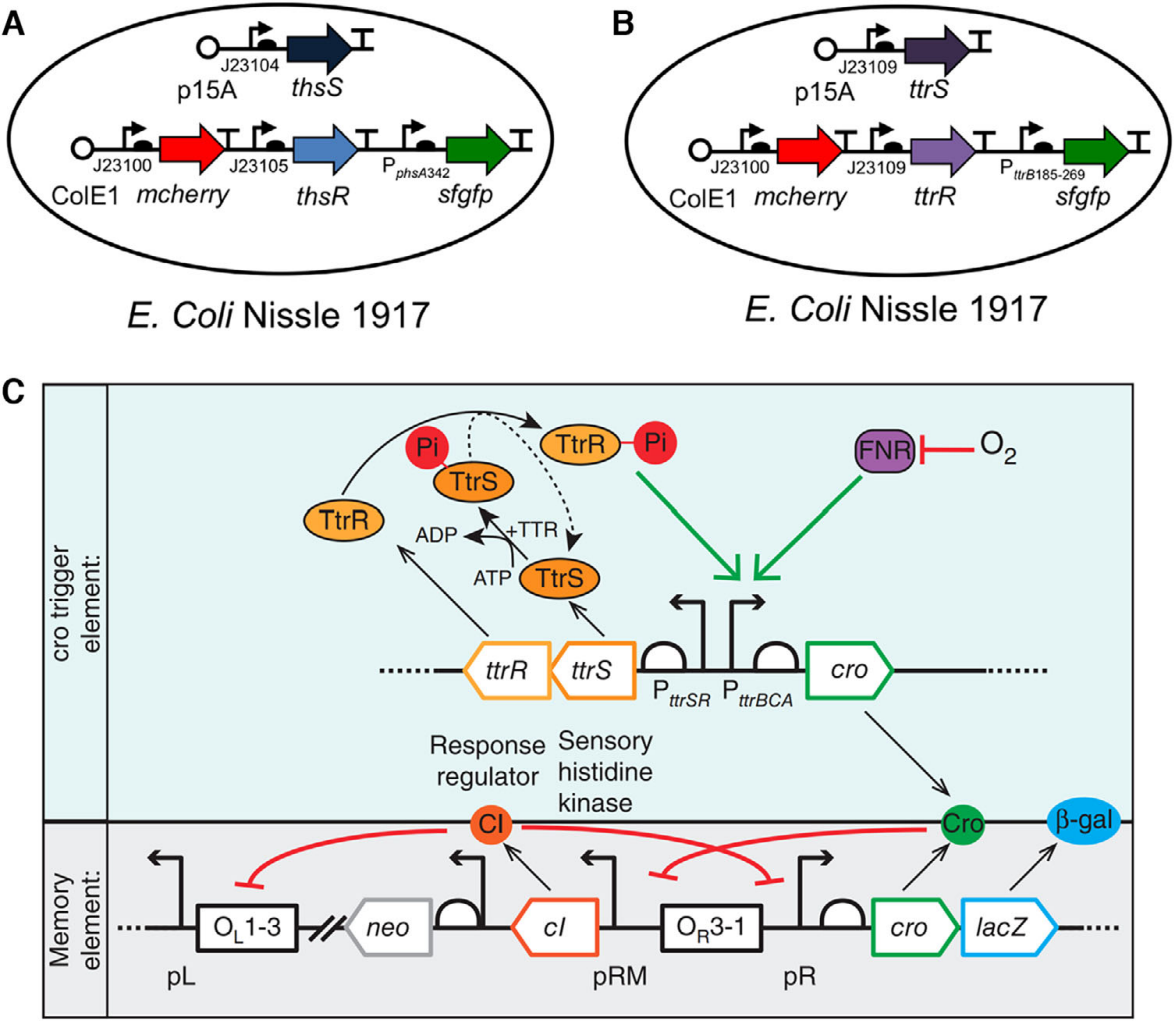
Construction of thiosulfate/tetrathionate sensor. Schematic of (A) thiosulfate and (B) tetrathionate sensor components. Reproduced under the terms of the Creative Commons CC BY license.^[^ ^93^ ^]^ Copyright 2017, Daeffler et al. (C) Components compositions and cro circuits triggered element and memory element. Reproduced with permission.^[^ ^95^ ^]^ Copyright 2017, Springer Nature.

Thiosulfate (S_2_O_3_^2−^) and tetrathionate (S_4_O_6_^2−^) are biomarkers related to gut sulfur metabolism and inflammation.^[^ ^93^ ^]^ Tetrathionate is a transient production from thiosulfate via ROS oxidation, as demonstrated in a mouse model of *S. typhimurium* gut infection.^[^ ^94^ ^]^ Therefore, Daeffler et al. used thiosulfate and tetrathionate as markers of intestinal inflammation.^[^ ^93^ ^]^ They discovered the first biological thiosulfate sensor (ThsSR) and an improved tetrathionate sensor (TtrSR) in the marine *Shewanella* species, which then were separately constructed with fluorescent reporter gene super folder green‐fluorescent reporter protein (sfGFP) and encoded into probiotic *E. coli* for mice gavage (Figure 5A,B). In the DSS‐induced colitis mice model, the engineered *E. coli* with ThsSR expressed higher sfGFP that could be detected by flow cytometry after excretion with feces, while *E. coli* with TtrSR did not show distinguishing sfGFP expression.^[^ ^93^ ^]^ Riglar's teams used the TtrSR and the promoter from *S. typhimurium* to drive the expression of Cro, which triggers the production of β‐galactosidase, an enzyme that causes bacterial clones to appear blue on plates containing 5‐bromo‐4‐chloro‐3‐indolyl‐d‐galactopyranoside (Figure 5C). In an *S. typhimurium*‐induced colitis mouse model, the presence of tetrasulfate can be detected by the sensor effectively for up to 6 months without genetic mutations.^[^ ^95^ ^]^

Chandrakasan et al. facilitated the diagnosis process by combining bacteria with electronic chips to convert the bacteria's responses into wireless communications.^[^ ^96^ ^]^ They designed a genetic circuit that enables the bacteria to glow when they sense heme. The bacteria were placed in a designed electronic capsule, covered by a membrane that is semipermeable to small molecules. A phototransistor beneath the bacteria monitored the amount of light emitted by the bacteria and transferred the data to a microprocessor, which then sent it via wireless signals to a nearby computer or smartphone. It is feasible to replace heme with thiosulfate or specific bacterial signal molecules,^[^ ^96^ ^]^ implying that this device might be duplicated in a variety of signaling molecule detections.

**FIGURE 6:**
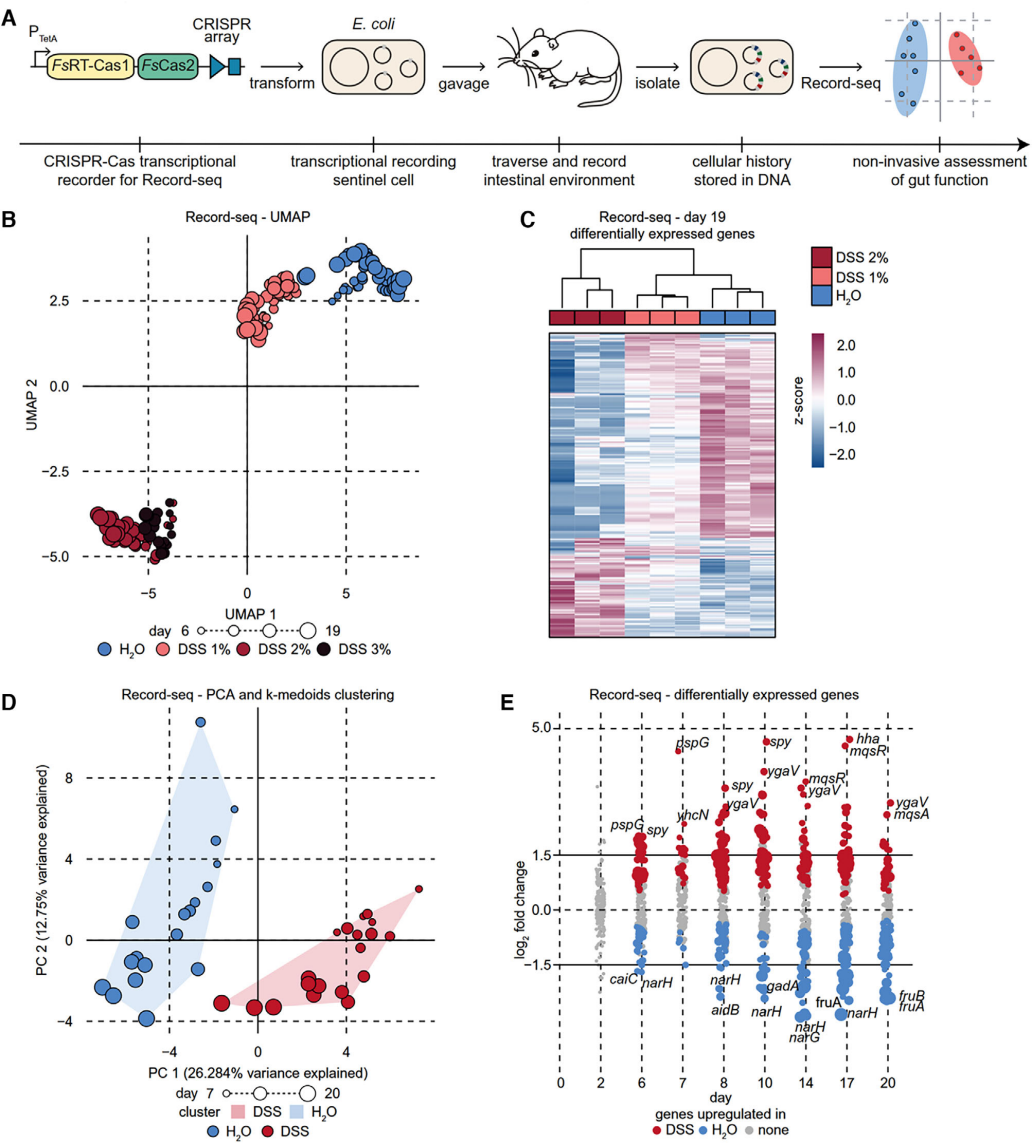
Sentinel cells record transcriptional information of gut. (A) Schematic of workflow for in vivo research with transcriptional recording sentinel cells. (B) Record‐seq data from mice treated with water or 1%, 2%, or 3% dextran sodium sulfate (DSS) were visualized and analyzed by uniform manifold approximation and projection (UMAP). (C) Hierarchical clustering of Record‐seq differentially expressed genes (DEGs) from mice treated with water or 1% or 2% DSS on day 19 in the heatmap. (D) Principal component analysis (PCA)‐projected Record‐seq data for mice treated with water or 2% DSS. Convex hulls indicate K‐medoids clusters. (E) Dot plot displaying log_2_FC for Record‐seq DEGs detected in mice treated with water or 2% DSS. The sizes of the dots increase with significance (*P*_adj_ range from 9.8 × 10^−19^ to 1.0). Reproduced with permission.^[^ ^97^ ^]^ Copyright 2022, The American Association for the Advancement of Science.

Further Mimee et al. monitored the intestinal information in detail by using a novel technique called Record‐seq which could record the gene expression of gut bacteria under different conditions, such as diet, inflammation, and microbial interactions.^[^ ^97^ ^]^ Record‐seq is based on CRISPR‐Cas, a natural immune system of bacteria that can capture snippets of RNA and store them as DNA in CRISPR arrays. The authors engineered *E*. *coli* sentinel cells to carry a CRISPR array and a reverse transcriptase Cas1‐Cas2 complex that can convert RNA into DNA and integrate it into the array. By sequencing the CRISPR arrays from fecal samples, the authors could reconstruct the history of gene expression in the sentinel cells and infer the environmental changes that occurred in the gut (Figure 6A). For example, the engineered *E*. *coli* distinguished different concentrations DSS‐treated and control mice and accurately reported on the phenotypic severity of the colitis model (Figure 6B,C), as well as revealing multiple features of the inflammatory environment by analyzing differentially expressed genes (DEGs), such as reduced anaerobic metabolism, increased oxidative and membrane stress, and nutrient limitation (Figure 6D,E).

Fecal calprotectin is extensively employed as a biomarker for diagnosing IBD and monitoring disease activity in clinical settings. Xia et al. conducted a screen for highly upregulated promotors in EcN when it was exposed to calprotectin. They then coupled these identified candidate genes to sfGFP to pinpoint a potential calprotectin biosensor—a zinc‐responsive promotor known as ykgMO. The researchers harnessed this calprotectin‐responsive gene to engineer a bioluminescent calprotectin‐sensing EcN (CS EcN), which could detect gut inflammation in mice through live animal luminescence imaging or fecal analysis. Importantly, CS EcN demonstrated the ability to differentiate between stool samples from individuals with active IBD and those in remission.^[^ ^98^ ^]^ The design of bacterial biosensors with key clinical indicators greatly enhances the potential for the application of engineered bacteria for IBD diagnosis.

## 5 DISCUSSION AND OUTLOOK

In this review, we have provided a comprehensive overview of bacterial‐based platforms for the treatment and diagnosis of IBD. Bacteria exhibit a distinctive ability to transverse the gastrointestinal tract and establish gut colonization, thereby facilitating their oral administration and enabling sustained and targeted therapeutic effects. Further understanding of the underlying mechanisms of IBD and the identification of novel therapeutic agents have facilitated the ongoing advancement of drug‐delivery bacteria. This innovative strategy shows substantial potential in mitigating systemic adverse effects and facilitating the management of IBD. Engineered bacteria can generate bioactive at different scales to treat intestinal inflammation according to multiple mechanisms involved in IBD pathogenesis, including immune dysregulation, oxidative stress, barrier damage, and microbiota dysbiosis. In addition, bacteria‐based platforms offer a less invasive and more timely strategy for acquiring intestinal information. By integrating therapeutic secretion and inflammation biomarker‐sensing capabilities, engineered bacteria exhibit the potential to treat intestinal lesions reactively and intelligently.

Scientists select bacteria‐based platforms according to their safety, capacity to survive in the gastrointestinal tract, genetic editability, and a stable and controlled level of drug production. Because of worries regarding human safety and the discharge of genetically modified organisms into the environment, the clinical use of genetically engineered bacteria is controversial. Despite the absence of significant mutations in the engineered bacteria during a 6‐month monitoring study,^[^ ^95^ ^]^ the long‐term implications remain uncertain. In addition to utilizing non‐modified bacteria for drug delivery, such as coating probiotic bacteria with adhesive nanomedicines,^[^ ^99^ ^]^ here are a few more strategies to consider. Fang et al. constructed a disposable engineered bacteria by integrating a self‐lysis system into the bacteria, enabling the concurrent processes of drug release and destruction of the modified bacteria.^[^ ^100^ ^]^ Thymineless death is a similar counter‐strategy for addressing engineered bacteria in vivo once their tasks are completed.^[^ ^101^ ^]^ After replacing the thymidylate synthase gene, the bacteria cannot reproduce if the thymus is not supplied or expelled from the body.^[^ ^102^ ^]^ However, research has found that *Bacteroides ovatus* could overcome the thymineless state by exchanging genetic material.^[^ ^103^ ^]^ SimCell, a synthetic bacterium with a degraded chromosome, only has genes for glycolysis and executing specific tasks. SimCell can express the synthetic genetic circuit continuously for up to 10 days, but they cannot be preserved for a long time.^[^ ^104^ ^]^ Despite this, there are still worries about ingesting foreign bacteria into the body. OMVs are non‐contagious and have little antigenicity, making them safer than bacteria.^[^ ^81^ ^]^ Besides, the asymmetric bacterial division produces chromosome‐free 200 to 400 nm vesicles known as minicells,^[^ ^105^ ^]^ which are currently commonly used for tumor medication delivery,^[^ ^106^ ^]^ but have not been explored for the treatment of IBD. OMVs and minicells are both bacterial‐derived, chromosome‐free, non‐transmissible novel vectors. OMVs retain the delivery capability of bacteria to load protein, DNA, and RNA while they cannot proliferate in the human body, which increases their safety as an IBD‐targeted platform.^[^ ^107^ ^]^

Numerous studies have underscored the positive impact of recombinant bacteria treatment on gut flora. In the experiment where mice were pretreated with antibiotics prior to the administration of the engineered bacterium ECN‐pE(C/A)2, a diminished efficacy against colitis was observed, suggesting a significant association between intestinal flora and disease improvement.^[^ ^61^ ^]^ Subsequent investigation revealed that ECN‐pE(C/A)2, producing SOD and CAT, enhanced the abundance and diversity of the flora and increased the levels of butyric acid‐producing probiotics, whereas unmodified EcN demonstrated did not significantly alter the gut flora. When studies utilize *L*. *lactis*, which has been shown to be beneficial to the intestinal flora, as carriers, probiotics carrying empty‐load plasmids induced some flora modulation, though their effectiveness was not comparable to engineered bacteria capable of drug production.^[^ ^50^, ^67^ ^]^ Therefore, the impact of engineered bacteria on intestinal flora appears to hinge on both the bacteria themselves and the drugs they deliver. Currently, no discernible role for gene editing has been identified. While existing research predominantly highlights the beneficial effects of engineered bacteria on gut flora, changes in small yet crucial microbial species might be missed and the potential adverse effects of gene editing on microbiota are unclear. Notably, most bacterial gene editing occurs on plasmids; however, free plasmids in bacteria may be transferred horizontally to other bacteria,^[^ ^103^ ^]^ so maybe it is safer to transfer gene fragments to the genome. It is imperative to meticulously investigate the long‐term effects of administering engineered bacteria to gut microbes.

Bacteria can deliver drugs to the gut and reduce systemic adverse events considerably. However, the gut‐targeting capacity of bacteria still needs further investigation. EcN, for example, was discovered to colonize primarily in the cecum and proximal colon, whereas UC is typically most severe near the rectum and CD can occur anywhere in the gastrointestinal tract. We hope that in the future, bacteria‐based platforms could be engineered to target the foci more precisely, possibly by targeting specific molecules in intestinal foci or disease‐associated pathogens.

Notably, Yu et al. explored the effects of EcN engineered to produce EGF (EGF‐EcN) in a mouse model of colitis‐associated cancer (CAC).^[^ ^64^ ^]^ Remarkably, EGF‐EcN administration led to a significant reduction in both the number and size of tumors, curbed cell proliferation within the tumor mass, and diminished high‐grade dysplasia in the mucosa. EGF is typically associated with promoting tumorigenesis and development. The surprising therapeutic effect of EGF‐EcN in CAC is likely due to its role in fortifying the intestinal barrier and lessening inflammation. Given the tumor‐targeted nature of bacteria^[^ ^35a^ ^]^ and the common occurrence of intestinal dysbiosis in CAC patients,^[^ ^108^ ^]^ this exploration into bacteria‐based platforms presents a highly promising avenue in CAC treatment.

The pathogenic mechanism of IBD is under active research. When the mechanisms of IBD are further revealed, bacteria will become better diagnostic tools in the future. In addition to identifying IBD markers, it may also be possible to identify IBD‐related genes or recognize IBD based on the flora characteristics. Engineered bacteria have the potential to become a novel gut diagnostic tool in a more sensitive, convenient, and noninvasive way. The potential for self‐testing of intestinal inflammation represents an exciting opportunity. To take a step further, engineered bacteria could be equipped with multifunctional capabilities of disease diagnosis and treatment simultaneously, thus greatly facilitating their therapeutic capacities in treating IBD. Scott et al. have successfully engineered yeast‐based probiotics that express a human P2Y2 purinergic receptor, resulting in a significant enhancement in sensitivity to extracellular adenosine triphosphate (eATP), a molecule known to induce intestinal inflammation.^[^ ^109^ ^]^ They linked the receptor activation to the secretion of apyrase, an enzyme responsible for ATP degradation. Consequently, the engineered yeast probiotics possessed the ability to sense a proinflammatory molecule and subsequently produce a self‐regulated response in proportion to the stimulus. These self‐tunable yeast probiotics reduced intestinal inflammation, fibrosis, and dysbiosis in mice models of IBD, while achieving efficacy comparable to or greater than standard‐of‐care therapies. Recently, Zou et al. integrated ThsSR, fluorescent reporter gene, CRISPR‐based genome editing technology, and therapeutic protein secretion system, obtaining a multifunction bacterium, that integrates the function of diagnosis, record, and treatment.^[^ ^110^ ^]^ The ThsSR sensor detected the presence of the intestinal inflammatory molecular thiosulfate, leading to the activation of downstream components such as the second‐generation base editor (BE2), the fluorescent reporter genes sfGFP and mCherry, as well as the α‐hemolysin‐secreting system for the secretion of the immunomodulator (AvCystatin). In a mouse model of DSS‐induced colitis, the engineered bacteria were able to diagnose early‐stage inflammations (DAI ≤ 5) by analyzing the fluorescence, base‐editing rate, and blue clone ratio in stool samples and colon content. Moreover, the production of the therapeutic protein AvCystatin in EcN is correlated with the degree of colon inflammations, resulting in a notable alleviation of colitis. This self‐tunable mechanism of drug secretion holds the potential to safeguard the intestine against the adverse effects associated with elevated drug concentrations, such as fibrosis. With the development of bioimaging and probes,^[^ ^111^ ^]^ the status of IBD can be more easily and consistently detected and treated in a timely and reactive manner according to the condition of the intestine by combining the functions of disease perception and treatment on intelligent therapeutic bacteria, simplifying the management of long‐term recurrent disease.

## AUTHOR CONTRIBUTIONS

Conceptualization: Jiaoying Lu and Juan Du. Writing—original draft preparation: Jiaoying Lu. Writing—review and editing: Xinyuan Shen, Jiaoying Lu, and Hongjun Li. Visualization: Xinyuan Shen. Supervision and project administration: Juan Du and Hongjun Li. Funding acquisition: Juan Du. All authors have read and agreed to the published version of the manuscript.

## CONFLICT OF INTEREST STATEMENT

The authors declare no conflicts of interest.

## References

[1] S. C. Ng , C. N. Bernstein , M. H. Vatn , P. L. Lakatos , E. V. Loftus Jr. , C. Tysk , C. O'Morain , B. Moum , J. F. Colombel , Gut 2013, 62, 630. 23335431 10.1136/gutjnl-2012-303661

[2] G. P. Ramos , K. A. Papadakis , Mayo Clin. Proc. 2019, 94, 155. 30611442 10.1016/j.mayocp.2018.09.013 PMC6386158

[3] I. Loddo , C. Romano , Front. Immunol. 2015, 6, 551. 26579126 10.3389/fimmu.2015.00551 PMC4629465

[4] a) A. N. Ananthakrishnan , H. Khalili , G. G. Konijeti , L. M. Higuchi , P. de Silva , J. R. Korzenik , C. S. Fuchs , W. C. Willett , J. M. Richter , A. T. Chan , Gastroenterology 2013, 145, 970; 23912083 10.1053/j.gastro.2013.07.050 PMC3805714

[5] a) N. Shi , N. Li , X. Duan , H. Niu , Mil. Med. Res. 2017, 4, 14; 28465831 10.1186/s40779-017-0122-9 PMC5408367

[6] J. Lloyd‐Price , C. Arze , A. N. Ananthakrishnan , M. Schirmer , J. Avila‐Pacheco , T. W. Poon , E. Andrews , N. J. Ajami , K. S. Bonham , C. J. Brislawn , D. Casero , H. Courtney , A. Gonzalez , T. G. Graeber , A. B. Hall , K. Lake , C. J. Landers , H. Mallick , D. R. Plichta , M. Prasad , G. Rahnavard , J. Sauk , D. Shungin , Y. Vázquez‐Baeza , R. A. White , 3rd, J. Braun , L. A. Denson , J. K. Jansson , R. Knight , S. Kugathasan , D. P. B. McGovern , J. F. Petrosino , T. S. Stappenbeck , H. S. Winter , C. B. Clish , E. A. Franzosa , H. Vlamakis , R. J. Xavier , C. Huttenhower , Nature 2019, 569, 655. 31142855 10.1038/s41586-019-1237-9 PMC6650278

[7] B. Yilmaz , P. Juillerat , O. Øyås , C. Ramon , F. D. Bravo , Y. Franc , N. Fournier , P. Michetti , C. Mueller , M. Geuking , V. E. H. Pittet , M. H. Maillard , G. Rogler , R. Wiest , J. Stelling , A. J. Macpherson , Nat. Med. 2019, 25, 323. 30664783 10.1038/s41591-018-0308-z

[8] J. Gubatan , D. R. Holman , C. J. Puntasecca , D. Polevoi , S. J. Rubin , S. Rogalla , World J. Gastroenterol. 2021, 27, 7402. 34887639 10.3748/wjg.v27.i43.7402 PMC8613745

[9] M. E. Johansson , J. K. Gustafsson , J. Holmén‐Larsson , K. S. Jabbar , L. Xia , H. Xu , F. K. Ghishan , F. A. Carvalho , A. T. Gewirtz , H. Sjövall , G. C. Hansson , Gut 2014, 63, 281. 23426893 10.1136/gutjnl-2012-303207 PMC3740207

[10] J. Landy , E. Ronde , N. English , S. K. Clark , A. L. Hart , S. C. Knight , P. J. Ciclitira , H. O. Al‐Hassi , World J. Gastroenterol. 2016, 22, 3117. 27003989 10.3748/wjg.v22.i11.3117 PMC4789987

[11] R. Gomez‐Bris , A. Saez , B. Herrero‐Fernandez , C. Rius , H. Sanchez‐Martinez , J. M. Gonzalez‐Granado , Int. J. Mol. Sci. 2023, 24, 2696. 36769019 10.3390/ijms24032696 PMC9916759

[12] Q. Guan , J. Zhang , Mediat. Inflamm. 2017, 2017, 4810258. 10.1155/2017/4810258 PMC5379128 28420941

[13] A. Geremia , P. Biancheri , P. Allan , G. R. Corazza , A. Di Sabatino , Autoimmun. Rev. 2014, 13, 3. 23774107 10.1016/j.autrev.2013.06.004

[14] M. F. Neurath , Nat. Rev. Immunol. 2014, 14, 329. 24751956 10.1038/nri3661

[15] a) W. Reinisch , D. W. Hommes , G. Van Assche , J. F. Colombel , J. P. Gendre , B. Oldenburg , A. Teml , K. Geboes , H. Ding , L. Zhang , M. Tang , M. Cheng , S. J. van Deventer , P. Rutgeerts , T. Pearce , Gut 2006, 55, 1138; 16492717 10.1136/gut.2005.079434 PMC1856289

[16] C. N. Bernstein , J. F. Blanchard , E. Kliewer , A. Wajda , Cancer 2001, 91, 854. 11241255 10.1002/1097-0142(20010215)91:4<854::aid-cncr1073>3.0.co;2-z

[17] a) K. W. Schroeder , W. J. Tremaine , D. M. Ilstrup , N. Engl. J. Med. 1987, 317, 1625; 3317057 10.1056/NEJM198712243172603

[18] H. Gordon , L. Biancone , G. Fiorino , K. H. Katsanos , U. Kopylov , E. Al Sulais , J. E. Axelrad , K. Balendran , J. Burisch , L. de Ridder , L. Derikx , P. Ellul , T. Greuter , M. Iacucci , C. Di Jiang , C. Kapizioni , K. Karmiris , J. Kirchgesner , D. Laharie , T. Lobatón , T. Molnár , N. M. Noor , R. Rao , S. Saibeni , M. Scharl , S. R. Vavricka , T. Raine , J. Crohns Colitis 2023, 17, 827. 36528797 10.1093/ecco-jcc/jjac187

[19] S. B. Hanauer , G. Stathopoulos , Drug Saf. 1991, 6, 192. 1676590 10.2165/00002018-199106030-00005

[20] H. Dai , Q. Fan , C. Wang , Exploration 2022, 2, 20210157. 37324799 10.1002/EXP.20210157 PMC10191059

[21] L. V. McFarland , Clin. Infect. Dis. 2015, 60(Suppl 2), S85.25922406

[22] L. V. McFarland , T. Karakan , A. Karatas , EClinicalMedicine 2021, 41, 101154. 34712929 10.1016/j.eclinm.2021.101154 PMC8529205

[23] a) J. Z. Goldenberg , L. Lytvyn , J. Steurich , P. Parkin , S. Mahant , B. C. Johnston , Cochrane Database Syst. Rev. 2015, 22, Cd004827; 10.1002/14651858.CD004827.pub4 26695080

[24] E. Dimidi , S. Christodoulides , K. C. Fragkos , S. M. Scott , K. Whelan , Am. J. Clin. Nutr. 2014, 100, 1075. 25099542 10.3945/ajcn.114.089151

[25] L. Kaur , M. Gordon , P. A. Baines , Z. Iheozor‐Ejiofor , V. Sinopoulou , A. K. Akobeng , Cochrane Database Syst. Rev. 2020, 3, Cd005573. 32128795 10.1002/14651858.CD005573.pub3 PMC7059959

[26] L. Steidler , W. Hans , L. Schotte , S. Neirynck , F. Obermeier , W. Falk , W. Fiers , E. Remaut , Science 2000, 289, 1352. 10958782 10.1126/science.289.5483.1352

[27] J. Ouyang , Z. Zhang , B. Deng , J. Liu , L. Wang , H. Liu , S. Koo , S. Chen , Y. Li , A. V. Yaremenko , X. Huang , W. Chen , Y. Lee , W. Tao , Mater. Today 2023, 62, 296.

[28] M. Elli , M. L. Callegari , S. Ferrari , E. Bessi , D. Cattivelli , S. Soldi , L. Morelli , N. G. Feuillerat , J. M. Antoine , Appl. Environ. Microbiol. 2006, 72, 5113. 16820518 10.1128/AEM.02950-05 PMC1489325

[29] P. L. Conway , S. L. Gorbach , B. R. Goldin , J. Dairy Sci. 1987, 70, 1. 10.3168/jds.S0022-0302(87)79974-3 3106442

[30] a) P. Lund , A. Tramonti , D. De Biase , FEMS Microbiol. Rev. 2014, 38, 1091; 24898062 10.1111/1574-6976.12076

[31] S. Li , W. Jiang , C. Zheng , D. Shao , Y. Liu , S. Huang , J. Han , J. Ding , Y. Tao , M. Li , J. Controlled Release 2020, 327, 801. 10.1016/j.jconrel.2020.09.011 32926886

[32] a) J. M. de Barros , T. Lechner , D. Charalampopoulos , V. V. Khutoryanskiy , A. D. Edwards , Int. J. Pharm. 2015, 493, 483; 26188314 10.1016/j.ijpharm.2015.06.051

[33] a) H. J. Park , G. H. Lee , J. Jun , M. Son , M. J. Kang , Drug Des., Dev. Ther. 2016, 10, 2599; 10.2147/DDDT.S103894 PMC4827893 27103789

[34] D. T. Riglar , P. A. Silver , Nat. Rev. Microbiol. 2018, 16, 214. 29398705 10.1038/nrmicro.2017.172

[35] a) M. Divyashree , S. K. Prakash , V. Aditya , A. A. A. Aljabali , K. J. Alzahrani , V. Azevedo , A. Goes‐Neto , M. M. Tambuwala , D. Barh , Future Oncol. 2022, 18, 1609; 35137604 10.2217/fon-2021-1137

[36] N. Qiao , G. Du , X. Zhong , X. Sun , Exploration 2021, 1, 20210026. 37323212 10.1002/EXP.20210026 PMC10191043

[37] a) E. Quévrain , M. A. Maubert , C. Michon , F. Chain , R. Marquant , J. Tailhades , S. Miquel , L. Carlier , L. G. Bermúdez‐Humarán , B. Pigneur , O. Lequin , P. Kharrat , G. Thomas , D. Rainteau , C. Aubry , N. Breyner , C. Afonso , S. Lavielle , J. P. Grill , G. Chassaing , J. M. Chatel , G. Trugnan , R. Xavier , P. Langella , H. Sokol , P. Seksik , Gut 2016, 65, 415; 26045134 10.1136/gutjnl-2014-307649 PMC5136800

[38] D. D. Mater , G. Corthier , J. Clin. Gastroenterol. 2004, 38, S64. 15220661 10.1097/01.mcg.0000128930.37156.71

[39] U. Sonnenborn , FEMS Microbiol. Lett. 2016, 363, fnw212. 27619890 10.1093/femsle/fnw212

[40] Z. Zhao , S. Xu , W. Zhang , D. Wu , G. Yang , Food Funct. 2022, 13, 5914. 35583304 10.1039/d2fo00226d

[41] a) W. Kruis , P. Fric , J. Pokrotnieks , M. Lukás , B. Fixa , M. Kascák , M. A. Kamm , J. Weismueller , C. Beglinger , M. Stolte , C. Wolff , J. Schulze , Gut 2004, 53, 1617; 15479682 10.1136/gut.2003.037747 PMC1774300

[42] M. Liu , S. Li , Q. Zhang , Z. Xu , J. Wang , H. Sun , Int. Immunopharmacol. 2018, 57, 25. 29455070 10.1016/j.intimp.2018.02.004

[43] R. Gardlik , A. Bartonova , P. Celec , Int. J. Mol. Med. 2013, 32, 492. 23708293 10.3892/ijmm.2013.1388

[44] M. Ma , Z. Zhao , Q. Liang , H. Shen , Z. Zhao , Z. Chen , R. He , S. Feng , D. Cao , G. Gan , H. Ye , W. Qiu , J. Deng , F. Ming , J. Jia , C. Sun , J. Li , L. Zhang , Appl. Microbiol. Biotechnol. 2021, 105, 5973. 34396488 10.1007/s00253-021-11472-y

[45] a) M. Cui , G. Pang , T. Zhang , T. Sun , L. Zhang , R. Kang , X. Xue , H. Pan , C. Yang , X. Zhang , J. Chang , J. Liu , S. Zhang , H. Wang , ACS Nano 2021, 15, 7040; 33819424 10.1021/acsnano.1c00135

[46] M. L. Hanson , J. A. Hixon , W. Li , B. K. Felber , M. R. Anver , C. A. Stewart , B. M. Janelsins , S. K. Datta , W. Shen , M. H. McLean , S. K. Durum , Gastroenterology 2014, 146, 210. 24120477 10.1053/j.gastro.2013.09.060 PMC3920828

[47] a) J. Wang , M. Tian , W. Li , F. Hao , Appl. Microbiol. Biotechnol. 2019, 103, 7931; 31456001 10.1007/s00253-019-10094-9

[48] K. J. Chua , H. Ling , I. Y. Hwang , H. L. Lee , J. C. March , Y. S. Lee , M. W. Chang , ACS Biomater. Sci. Eng. 2023, 9, 5123. 36399014 10.1021/acsbiomaterials.2c00202 PMC10498420

[49] A. P. Arukha , C. F. Freguia , M. Mishra , J. K. Jha , S. Kariyawasam , N. A. Fanger , E. M. Zimmermann , G. R. Fanger , B. Sahay , Biomedicines 2021, 9, 1098. 34572293 10.3390/biomedicines9091098 PMC8470720

[50] L. Song , W. Xie , Z. Liu , D. Guo , D. Zhao , X. Qiao , L. Wang , H. Zhou , W. Cui , Y. Jiang , Y. Li , Y. Xu , L. Tang , Appl. Microbiol. Biotechnol. 2019, 103, 6169. 31165225 10.1007/s00253-019-09898-6

[51] L. Wang , Y. Liao , R. Yang , Z. Zhu , L. Zhang , Z. Wu , X. Sun , Bioeng. Transl. Med. 2021, 6, e10219. 34589596 10.1002/btm2.10219 PMC8459592

[52] X. Yan , X. Y. Liu , D. Zhang , Y. D. Zhang , Z. H. Li , X. Liu , F. Wu , G. Q. Chen , Cell. Mol. Immunol. 2021, 18, 2344. 34480146 10.1038/s41423-021-00760-2 PMC8484604

[53] K. Vandenbroucke , H. de Haard , E. Beirnaert , T. Dreier , M. Lauwereys , L. Huyck , J. Van Huysse , P. Demetter , L. Steidler , E. Remaut , C. Cuvelier , P. Rottiers , Mucosal Immunol. 2010, 3, 49. 19794409 10.1038/mi.2009.116

[54] a) A. N. Shkoporov , E. V. Khokhlova , K. A. Savochkin , L. I. Kafarskaia , B. A. Efimov , FEMS Microbiol. Lett. 2015, 362, fnv083; 25994292 10.1093/femsle/fnv083

[55] a) K. Skrlec , P. Zadravec , M. Hlavnickova , M. Kuchar , L. Vankova , H. Petrokova , L. Krizova , J. Cerny , A. Berlec , P. Maly , Int. J. Mol. Sci. 2018, 19, 1933; 29966384 10.3390/ijms19071933 PMC6073689

[56] A. Zahirović , A. Berlec , Microb. Cell Fact. 2022, 21, 143. 35842694 10.1186/s12934-022-01873-7 PMC9287920

[57] F. Namai , S. Shigemori , T. Ogita , T. Sato , T. Shimosato , Exp. Mol. Med. 2020, 52, 1627. 32989233 10.1038/s12276-020-00507-5 PMC7520878

[58] G. Esposito , M. Pesce , L. Seguella , J. Lu , C. Corpetti , A. Del Re , F. D. E. De Palma , G. Esposito , W. Sanseverino , G. Sarnelli , Int. J. Mol. Sci. 2021, 22, 14, 2945. 33799405 10.3390/ijms22062945 PMC7999950

[59] Y. T. Park , T. Kim , J. Ham , J. Choi , H. S. Lee , Y. J. Yeon , S. I. Choi , N. Kim , Y. R. Kim , Y. J. Seok , Microb. Biotechnol. 2022, 15, 832. 33729711 10.1111/1751-7915.13795 PMC8913873

[60] K. Škrlec , R. Ručman , E. Jarc , P. Sikirić , U. Švajger , T. Petan , M. Perišić Nanut , B. Štrukelj , A. Berlec , Appl. Microbiol. Biotechnol. 2018, 102, 10103. 30191288 10.1007/s00253-018-9333-6

[61] J. Zhou , M. Y. Li , Q. F. Chen , X. J. Li , L. F. Chen , Z. L. Dong , W. J. Zhu , Y. Yang , Z. Liu , Q. Chen , Nat. Commun. 2022, 13, 3432. 35701435 10.1038/s41467-022-31171-0 PMC9198027

[62] L. Ortiz‐Velez , A. Goodwin , L. Schaefer , R. A. Britton , Front. Bioeng. Biotechnol. 2020, 8, 543. 32582668 10.3389/fbioe.2020.00543 PMC7289926

[63] K. Vandenbroucke , W. Hans , J. Van Huysse , S. Neirynck , P. Demetter , E. Remaut , P. Rottiers , L. Steidler , Gastroenterology 2004, 127, 502. 15300583 10.1053/j.gastro.2004.05.020

[64] M. Yu , J. Kim , J. H. Ahn , Y. Moon , JCI Insight 2019, 4, e125166. 31434808 10.1172/jci.insight.125166 PMC6777819

[65] C. C. Wong , L. Zhang , W. K. Wu , J. Shen , R. L. Chan , L. Lu , W. Hu , M. X. Li , L. F. Li , S. X. Ren , Y. F. Li , J. Li , C. H. Cho , J. Gastroenterol. Hepatol. 2017, 32, 609. 27470075 10.1111/jgh.13499

[66] R. Carvalho , A. Vaz , F. L. Pereira , F. Dorella , E. Aguiar , J. M. Chatel , L. Bermudez , P. Langella , G. Fernandes , H. Figueiredo , A. Goes‐Neto , V. Azevedo , Sci. Rep. 2018, 8, 15072. 30305667 10.1038/s41598-018-33469-w PMC6180057

[67] H. Tian , J. Li , X. Chen , Z. Ren , X. Pan , W. Huang , M. Bhatia , L. L. Pan , J. Sun , J. Agric. Food Chem. 2023, 71, 5185. 36943701 10.1021/acs.jafc.2c07098

[68] L. Peyrin‐Biroulet , W. J. Sandborn , R. Panaccione , E. Domènech , L. Pouillon , B. Siegmund , S. Danese , S. Ghosh , Therap. Adv. Gastroenterol. 2021, 14, 175628482110599. 10.1177/17562848211059954 PMC8669878 34917173

[69] A. Berlec , M. Perse , M. Ravnikar , M. Lunder , A. Erman , A. Cerar , B. Strukelj , Int. Immunopharmacol. 2017, 43, 219. 28039805 10.1016/j.intimp.2016.12.027

[70] S. Ferenczi , N. Solymosi , I. Horvath , N. Szeocs , Z. Grozer , D. Kuti , B. Juhasz , Z. Winkler , T. Pankotai , F. Sukosd , A. Stagel , M. Paholcsek , D. Dora , N. Nagy , K. J. Kovacs , I. Zanoni , Z. Szallasi , Mol. Ther.–Methods Clin. Dev. 2021, 20, 218. 33426148 10.1016/j.omtm.2020.11.010 PMC7782194

[71] R. Russo , C. Cristiano , C. Avagliano , C. De Caro , G. La Rana , G. M. Raso , R. B. Canani , R. Meli , A. Calignano , Curr. Med. Chem. 2018, 25, 3930. 28215162 10.2174/0929867324666170216113756

[72] H. Liu , J. Wang , T. He , S. Becker , G. Zhang , D. Li , X. Ma , Adv. Nutr. 2018, 9, 21. 29438462 10.1093/advances/nmx009 PMC6333934

[73] a) H. Zhu , Y. R. Li , Exp. Biol. Med. 2012, 237, 474; 10.1258/ebm.2011.011358 22442342

[74] a) Y. Cao , K. Cheng , M. Yang , Z. Deng , Y. Ma , X. Yan , Y. Zhang , Z. Jia , J. Wang , K. Tu , J. Liang , M. Zhang , J. Nanobiotechnol. 2023, 21, 21; 10.1186/s12951-023-01770-0 PMC9854161 36658555

[75] G. Tang , J. He , J. Liu , X. Yan , K. Fan , Exploration 2021, 1, 75. 37366468 10.1002/EXP.20210005 PMC10291575

[76] C. I. van de Wetering , M. C. Coleman , D. R. Spitz , B. J. Smith , C. M. Knudson , Free Radic Biol. Med. 2008, 44, 1677. 18291119 10.1016/j.freeradbiomed.2008.01.022 PMC2374742

[77] A. Sturm , A. U. Dignass , World J. Gastroenterol. 2008, 14, 348. 18200658 10.3748/wjg.14.348 PMC2679124

[78] A. M. Duraj‐Thatte , N. D. Courchesne , P. Praveschotinunt , J. Rutledge , Y. Lee , J. M. Karp , N. S. Joshi , Adv. Mater. 2019, 31, e1901826. 31402514 10.1002/adma.201901826 PMC6773506

[79] P. Praveschotinunt , A. M. Duraj‐Thatte , I. Gelfat , F. Bahl , D. B. Chou , N. S. Joshi , Nat. Commun. 2019, 10, 5580. 31811125 10.1038/s41467-019-13336-6 PMC6898321

[80] A. Sinha , J. Nightingale , K. P. West , J. Berlanga‐Acosta , R. J. Playford , N. Engl. J. Med. 2003, 349, 350. 12878742 10.1056/NEJMoa013136

[81] a) C. Schwechheimer , M. J. Kuehn , Nat. Rev. Microbiol. 2015, 13, 605; 26373371 10.1038/nrmicro3525 PMC5308417

[82] M. Kaparakis‐Liaskos , R. L. Ferrero , Nat. Rev. Immunol. 2015, 15, 375. 25976515 10.1038/nri3837

[83] A. L. Carvalho , S. Fonseca , A. Miquel‐Clopés , K. Cross , K. S. Kok , U. Wegmann , K. Gil‐Cordoso , E. G. Bentley , S. H. M. Al Katy , J. L. Coombes , A. Kipar , R. Stentz , J. P. Stewart , S. R. Carding , J. Extracell. Vesicles 2019, 8, 1632100. 31275534 10.1080/20013078.2019.1632100 PMC6598475

[84] J. Shi , Z. Ma , H. Pan , Y. Liu , Y. Chu , J. Wang , L. Chen , J. Microencapsulation 2020, 37, 481. 32700606 10.1080/02652048.2020.1797914

[85] a) N. Pai , J. Popov , L. Hill , E. Hartung , K. Grzywacz , P. Moayyedi , Gastroenterology 2021, 161, 388; 33961887 10.1053/j.gastro.2021.04.067

[86] H. Fang , L. Fu , J. Wang , Biomed. Res. Int. 2018, 2018, 8941340. 30302341 10.1155/2018/8941340 PMC6158944

[87] C. Airola , A. Severino , S. Porcari , W. Fusco , B. H. Mullish , A. Gasbarrini , G. Cammarota , F. R. Ponziani , G. Ianiro , Antibiotics 2023, 12, 868. 37237771 10.3390/antibiotics12050868 PMC10215521

[88] M. Fischer , D. Kao , C. Kelly , A. Kuchipudi , S. M. Jafri , M. Blumenkehl , D. Rex , M. Mellow , N. Kaur , H. Sokol , G. Cook , M. J. Hamilton , E. Phelps , B. Sipe , H. Xu , J. R. Allegretti , Inflamm. Bowel Dis. 2016, 22, 2402. 27580384 10.1097/MIB.0000000000000908

[89] Z. DeFilipp , P. P. Bloom , M. T Soto , M. K. Mansour , M. R. A. Sater , M. H. Huntley , S. Turbett , R. T. Chung , Y. B. Chen , E. L. Hohmann , N. Engl. J. Med. 2019, 381, 2043. 31665575 10.1056/NEJMoa1910437

[90] C. C. Wong , L. Zhang , Z. J. Li , W. K. Wu , S. X. Ren , Y. C. Chen , T. B. Ng , C. H. Cho , J. Gastroenterol. Hepatol. 2012, 27, 1205. 22507188 10.1111/j.1440-1746.2012.07158.x

[91] N. M. Breyner , P. B. V. Boas , G. Fernandes , R. D. de Carvalho , T. Rochat , M. L. Michel , F. Chain , H. Sokol , M. de Azevedo , A. Myioshi , V. A. Azevedo , P. Langella , L. G. Bermúdez‐Humarán , J. M. Chatel , Environ. Microbiol. 2019, 21, 4020. 31325218 10.1111/1462-2920.14748 PMC6899824

[92] F. Ostadhossein , P. Moitra , N. Gunaseelan , M. Nelappana , C. Lowe , M. Moghiseh , A. Butler , N. de Ruiter , H. Mandalika , I. Tripathi , S. K. Misra , D. Pan , Nanoscale Horiz. 2022, 7, 533. 35311837 10.1039/d1nh00626f

[93] K. N. Daeffler , J. D. Galley , R. U. Sheth , L. C. Ortiz‐Velez , C. O. Bibb , N. F. Shroyer , R. A. Britton , J. J. Tabor , Mol. Syst. Biol. 2017, 13, 923. 28373240 10.15252/msb.20167416 PMC5408782

[94] S. E. Winter , P. Thiennimitr , M. G. Winter , B. P. Butler , D. L. Huseby , R. W. Crawford , J. M. Russell , C. L. Bevins , L. G. Adams , R. M. Tsolis , J. R. Roth , A. J. Bäumler , Nature 2010, 467, 426. 20864996 10.1038/nature09415 PMC2946174

[95] D. T. Riglar , T. W. Giessen , M. Baym , S. J. Kerns , M. J. Niederhuber , R. T. Bronson , J. W. Kotula , G. K. Gerber , J. C. Way , P. A. Silver , Nat. Biotechnol. 2017, 35, 653. 28553941 10.1038/nbt.3879 PMC5658125

[96] M. Mimee , P. Nadeau , A. Hayward , S. Carim , S. Flanagan , L. Jerger , J. Collins , S. McDonnell , R. Swartwout , R. J. Citorik , V. Bulović , R. Langer , G. Traverso , A. P. Chandrakasan , T. K. Lu , Science 2018, 360, 915. 29798884 10.1126/science.aas9315 PMC6430580

[97] F. Schmidt , J. Zimmermann , T. Tanna , R. Farouni , T. Conway , A. J. Macpherson , R. J. Platt , Science 2022, 376, eabm6038. 35549411 10.1126/science.abm6038 PMC11163514

[98] J. Y. Xia , C. Hepler , P. Tran , N. J. Waldeck , J. Bass , A. Prindle , Proc. Nat. Acad. Sci. U. S. A. 2023, 120, e2221121120. 10.1073/pnas.2221121120 PMC10410751 37523538

[99] J. J. Li , W. L. Hou , S. S. Lin , L. Wang , C. Pan , F. Wu , J. Y. Liu , Adv. Sci. 2022, 9, 2104006. 10.1002/advs.202104006 PMC8728836 34713621

[100] T. T. Fang , Z. P. Zou , Y. Zhou , B. C. Ye , ACS Synth. Biol. 2022, 11, 3004. 36037444 10.1021/acssynbio.2c00182

[101] L. Steidler , Discov. Med. 2003, 3, 49. 20705040

[102] A. P. Arukha , C. F. Freguia , M. Mishra , J. K. Jha , S. Kariyawasam , N. A. Fanger , E. M. Zimmermann , G. R. Fanger , B. Sahay , Biomedicines 2021, 9, 1098. 34572293 10.3390/biomedicines9091098 PMC8470720

[103] U. Wegmann , A. L. Carvalho , M. Stocks , S. R. Carding , Sci. Rep. 2017, 7, 2294. 28536456 10.1038/s41598-017-02591-6 PMC5442108

[104] C. Fan , P. A. Davison , R. Habgood , H. Zeng , C. M. Decker , M. G. Salazar , K. Lueangwattanapong , H. E. Townley , A. Yang , I. P. Thompson , H. Ye , Z. Cui , F. Schmidt , C. N. Hunter , W. E. Huang , Proc. Nat. Acad. Sci. U. S. A. 2020, 117, 6752. 10.1073/pnas.1918859117 PMC7104398 32144140

[105] J. A. MacDiarmid , N. B. Mugridge , J. C. Weiss , L. Phillips , A. L. Burn , R. P. Paulin , J. E. Haasdyk , K. A. Dickson , V. N. Brahmbhatt , S. T. Pattison , A. C. James , G. Al Bakri , R. C. Straw , B. Stillman , R. M. Graham , H. Brahmbhatt , Cancer Cell 2007, 11, 431. 17482133 10.1016/j.ccr.2007.03.012

[106] M. K. Ali , Q. Liu , K. Liang , P. Li , Q. Kong , Cancer Lett. 2020, 491, 11. 32721550 10.1016/j.canlet.2020.07.024

[107] L. J. Kubiatowicz , A. Mohapatra , N. Krishnan , R. H. Fang , L. Zhang , Exploration 2022, 2, 20210217. 36249890 10.1002/EXP.20210217 PMC9539018

[108] M. L. Richard , G. Liguori , B. Lamas , G. Brandi , G. da Costa , T. W. Hoffmann , M. P. Di Simone , C. Calabrese , G. Poggioli , P. Langella , M. Campieri , H. Sokol , Gut Microbes 2018, 9, 131. 28914591 10.1080/19490976.2017.1379637 PMC5989788

[109] B. M. Scott , C. Gutiérrez‐Vázquez , L. M. Sanmarco , J. A. da Silva Pereira , Z. Li , A. Plasencia , P. Hewson , L. M. Cox , M. O'Brien , S. K. Chen , P. M. Moraes‐Vieira , B. S. W. Chang , S. G. Peisajovich , F. J. Quintana , Nat. Med. 2021, 27, 1212. 34183837 10.1038/s41591-021-01390-x

[110] Z. P. Zou , Y. Du , T. T. Fang , Y. Zhou , B. C. Ye , Cell Host Microbe 2023, 31, 199. 36758520 10.1016/j.chom.2022.12.004

[111] a) K. Wanderi , Z. Cui , Exploration 2022, 2, 20210097; 37323884 10.1002/EXP.20210097 PMC10191020

